# How do risk preferences relate to malaria care-seeking behavior and the acceptability of a new health technology in Nigeria?

**DOI:** 10.1186/1472-6963-14-374

**Published:** 2014-09-05

**Authors:** Jenny Liu, Sepideh Modrek, Jennifer Anyanti, Ernest Nwokolo, Anna De La Cruz, Eric Schatzkin, Chinwoke Isiguzo, Chinazo Ujuju, Dominic Montagu

**Affiliations:** Global Health Sciences, University of California, San Francisco, 550 16th Street, Mission Hall: Global Health & Clinical Sciences Building, San Francisco, CA 94158 USA; General Medical Disciplines, Stanford University, 1070 Arastradero Road Rm 311, Palo Alto, CA 94304-1334 USA; Society for Family Health, No 8 Port Harcourt Crescent, Area 11 Garki, Abuja, Nigeria

**Keywords:** Malaria, Risk preferences, Health behavior, Care-seeking behavior, Rapid diagnostic tests, Nigeria

## Abstract

**Background:**

To reduce the burden of disease from malaria, innovative approaches are needed to engender behavior change. One unobservable, but fundamental trait—preferences for risk—may influence individuals’ willingness to adopt new health technologies. We explore the association of risk preferences with malaria care-seeking behavior and the acceptability of malaria rapid diagnostic tests (RDTs) to inform RDT scale-up plans.

**Methods:**

In Oyo State, Nigeria, adult customers purchasing anti-malarial medications at selected drug shops took surveys and received an RDT as they exited. After an initial risk preference assessment via a simple lottery game choice, individuals were given their RDT result and treatment advice, and called four days later to assess treatment adherence. We used bivariable and multivariable regression analysis to assess the association of risk game choices with malaria care-seeking behaviors and RDT acceptability.

**Results:**

Of 448 respondents, 63.2% chose the lottery game with zero variance in expected payout, 27.9% chose the game with low variance, and 8.9% chose the game with high variance. Compared to participants who chose lower variance games, individuals choosing higher variance games were older, less educated, more likely to be male, and were more likely to patronize lower quality drug shops, seek care immediately, and report complete disability due to their illness. In contrast, individuals choosing lower variance games were more likely to follow the correct treatment directions and were more likely to report an increase in their willingness to pay for an RDT compared to other risk groups, our two measures of RDT acceptability. Differences in estimated associations between risk game choices and selected care-seeking behaviors remained after controlling sociodemographic confounders.

**Conclusions:**

The uptake of health diagnostic information in terms of translating the RDT experience into willingness to pay for an RDT and treatment adherence to test results may vary according to risk preferences. Hence, health promotion communications may want to be crafted bearing in mind differences in uptake among people of different risk preferences to encourage wider RDT adoption and more rational malaria treatment. Estimates will serve as the basis for power calculations for an expanded study.

## Background

A number of new technologies offer great promise for improving health outcomes in developing countries, including deworming drugs, clean water systems, and cleaner burning stoves [1, 2]. However, adoption has been slow and long-term behavior change has proven difficult to sustain [3]. Prevailing preferences toward risk could offer one explanation for depressed uptake. For example, risk aversion, or the reluctance of a person to accept a situation with an uncertain payoff rather than one with a more certain but potentially lower payoff, may be a barrier to accepting beneficial technology. Risk-averse people may be more apt to follow past behaviors if trying an innovation involves accepting a level of uncertainty about the technology’s effectiveness. Unobserved traits and specific preferences are theorized to influence health-decision making (e.g. [4]) and recent empirical research has confirmed associations of risk attitudes with various health behaviors, including smoking, drinking, and using seat belts [5]. Risk aversion has also served as the basis for health behavior change interventions even if not explicitly measured, such as those that seek to help individuals to internalize and recognize risky sexual practices [6].

Despite the theoretical importance of risk preferences, little is known about its relation to the acceptability of new health technologies. This study is an initial investigation of the association of risk preferences with the acceptability of a new health technology—the malaria rapid diagnostic test (RDT). This is part of a larger pilot study of the quality of malaria diagnosis and treatment at for-profit drug vendors conducted in Oyo State, Nigeria.

Despite the scale-up of many malaria interventions [7], increasing uptake of new health products and sustaining behavior change remain challenging. Simply overcoming income constraints via subsidies and micro-loans have increased purchases of bed nets, artemisinin-based combination therapies (ACTs), and RDTs, but results for improving actual use of nets or treatment compliance with test results have been disappointing [8, 9].^a^ While ability to pay and socioeconomic status are important factors in determining malaria care-seeking behavior (e.g. [10, 11]), seeking other root causes should be further explored. Hence, understanding the fundamental drivers of health behaviors and the underpinnings of behavior change are important for developing appropriate intervention delivery mechanisms that enhance adoption and promote better prevention and treatment practices.

In sub-Saharan Africa, even though access to effective malaria treatment with ACTs has increased [12], access to reliable diagnostics is problematic, leading to concerns of over-treatment, drug resistance, and lack of appropriate treatment for non-malaria illnesses [13]. The malaria RDT is a reliable method for diagnosing uncomplicated malaria and a cheap alternative to blood slide microscopy [14]. However, uptake and adherence remain challenging. Even among trained providers, trust and acceptability of RDT results are difficult to instill when clinical experience in symptomatic diagnosis signal contradictory indications [15]. Yet, acceptability has improved over time, resulting in cost-savings from less over-treatment [16–19].

In Nigeria, nearly 60% of people with suspected malaria seek care from private sector drug shops [20]. RDTs are currently being rolled out in the public healthcare sector [21], but more guidance on private sector delivery is needed before expanding access. A recent nationwide survey indicates that 14% of public and not-for-profit facilities stocked RDTs, but only 1.4% of private sector outlets [22]. In addition, current regulatory guidelines prohibit drug shops from performing diagnostic tests even though they are the main sources for antimalarial drugs. In other countries, provision of RDTs via private sector channels has been more problematic than public sector implementation and initial studies show that high subsidies were necessary to foster uptake, but that patient compliance to test results was generally poor.^b^

Further, little consumer demand exists for malaria diagnosis given the wide availability of cheap anti-malarial drugs; presumptive treatment is still the norm [23, 24]. Although RDTs may improve the quality of malaria case management, nothing is known about the acceptability of RDTs and the adherence to treatment among customers at private sector drug shops in Nigeria. To stem presumptive treatment and improve appropriate care for acute malaria episodes, sick individuals must become comfortable with a new care protocol: they must accept testing before treatment, believe in the test results, and take the correct treatment.

We hypothesize that risk preferences among sick individuals influence care-seeking behavior and are associated with the acceptability and valuation of RDTs. Because the RDT is a health technology that confers information about health status, it is *a priori* unclear how individuals of different risk preferences may perceive RDTs. This is because there are two types of uncertainties involved in the use of an RDT—one questioning the validity of the RDT device itself and one regarding a person’s malaria status—and individuals may prefer to reduce one uncertainty over the other. On the one hand, risk averse individuals may be hesitant to adopt and listen to a new technology if they feel uncertain about the integrity of the device, preferring to presumptively self-treat based on prior experiences. On the other hand, the introduction of diagnostic information reduces the uncertainty of having the suspected illness and risk averse individuals may express more affinity for the test due to the elimination of disease status uncertainty.

In this exploratory study, we investigate the directionality of this relationship and the influence of other sociodemographic confounders through which risk preferences may influence health behaviors and the uptake of diagnostic information. From a sample of adult customers with anti-malarial medication purchases from private drug shops in Nigeria who agreed to be surveyed and take a free RDT, we analyzed their self-reported care-seeking and treatment behaviors and RDT acceptability in relation to their choice in a simple lottery game designed to assess risk preferences.

## Methods

### Study area and sample selection

This study took place in urban and peri-urban areas of Oyo State in southwest Nigeria, in and around the cities of Ibadan and Ogbomosho. The population is predominantly of Yoruba descent and all study survey data collectors and nurses were fluent in both English and the Yoruba language. Oyo state is holoendemic for malaria; over 50% of the population have one attack per year and children under five experience two to four episodes per year [20].

Drug shops were chosen as the best site for participant recruitment because most individuals in Nigeria obtain malaria treatment from these vendors [20]. From all enumerated drug shops within four selected local government areas, a total of 50 shops—pharmacies or proprietary and patent medicine vendors (PPMVs)—were stratified by type and randomly selected, 43 of which agreed to participate in the study and to serve as enrollment sites (21 pharmacies and 23 PPMVs). In October 2012, two members of the survey team, one trained nurse and one researcher, were stationed at enrolled shops on randomly selected days of the week (excluding Sunday) and approached customers as they exited the drug store to assess eligibility. Participants were eligible for the study if they were an adult (aged 18 or over), not pregnant, had just purchased treatment for malaria for him- or herself, and would be willing to have an RDT conducted for free. Individuals purchasing malaria treatment for others were excluded. During screening, participants were made aware that they would be compensated for their time with a small mobile phone credit of 100 Naira (~US$0.63) paid at the end of the interview and would be invited to earn more based on the outcome of a game (risk preference game detailed below). All compensation amounts were vetted and pre-tested with local experts to determine feasible amounts that would minimize undue influence on an individual’s willingness to participate.

### Data collection

For eligible participants who signed written consent to participate, two surveys were conducted—one at the time of enrollment and testing (i.e. baseline) and one four days after the initial encounter via phone (i.e. follow-up). At baseline, the eligible participant was given a RDT (free of charge) by a trained nurse at the beginning of the survey. While the RDT result was pending (about 15 minutes), a short survey was administered, including an inventory of drugs purchased. Contact information was recorded to enable later phone follow-up. At the end of the survey, the participant was provided with the result of his or her test.

Nurses were instructed to provide participants with standard advice according to their RDT results. If the participant tested positive for malaria, s/he was told that the positive result indicated the presence of malaria. Per ethical considerations and to ensure that the participants testing positive had a quality-assured anti-malaria drug, a free course of ACTs was provided and participants were instructed to take the quality-assured ACT instead of the anti-malarial drugs s/he purchased on her/his own. If the test was negative, the participant was told that the negative result indicated the absence of malaria and that the anti-malarial drugs they purchased were not needed. Regardless of the test result, all participants were referred to local clinics and hospitals where they could seek care if their condition was not malaria, or if their illness became worse. In a parallel study of a randomized intervention to influence RDT adherence, selected participants were sent a SMS text message reminder of the RDT results one day after the baseline survey [25]. This component of the study was unrelated to the risk preference assessment, and analyses show no relationship of the SMS intervention to risk game choices analyzed here [25].

Four days after the baseline survey, a study nurse called each participant and conducted a phone interview to obtain self-reported information on the state of their health and the drugs they had used. A total of 465 adults were enrolled in the baseline survey, but eight were excluded due to survey numbering errors, and 424 participated in the follow-up phone survey. Less than 4% of participants were RDT-positive among all enrolled. Because of the exploratory nature of the risk preference analysis, no power calculations were conducted to *a priori* determine a target sample size.

### Measuring health behaviors and RDT acceptability

During the baseline survey, we asked participants about their usual and current care-seeking behavior for malaria: having ever had a blood test, usual diagnostic method, severity of illness the last time s/he had malaria, type of provider seen for the last suspected episode, the number of days waited before seeking care, source of their diagnosis, and their drug purchases.

To assess acceptability and valuation of RDTs, we used two measures. First, we asked respondents two rounds of willingness to pay questions—once during the baseline survey after participants received their RDT result while individuals were feeling sick and again during the phone follow-up survey after individuals should have recovered from their illness. We modified contingent valuation methods in other RDT valuation studies [24, 26] and used a bidding approach: starting with a market price of 200 Naira (~US$1.20) and depending on the respondent’s answer, the amount was marked down by 50 Naira increments with a final bid of 20 Naira before a floor of zero was reached, or marked up by 50 Naira with a final bid of 400 Naira before a ceiling of 500 Naira was reached. Respondents could bid as many times as they desired and their last bid was taken to represent their willingness to pay. Because RDTs are not widely available and few respondents had previously experienced an RDT, a comparison of the two measures of willingness to pay enables us to examine *changes* in valuation rather than levels, which may be inaccurate and confounded by past experiences or individuals’ priors with diagnostic procedures. The *increase* in willingness to pay for an RDT is arguably a more accurate reflection of the participants’ acceptability of the RDT and is purposely designed to allow individuals to have time to learn the value of the RDT over the course of the illness recovery. Second, treatment adherence to RDT results, an indirect measure of the value of diagnostic information, was determined through the follow-up phone survey in which nurses asked respondents about drugs taken. We considered a respondent to have followed the treatment directions if s/he took the anti-malarial when RDT-positive or if s/he did not take the anti-malarial when RDT-negative.

### Measuring risk preferences

We assessed risk preferences using a simplified lottery game choice set, which was part of the baseline survey. Respondents chose one lottery game to play amongst three game options: (1) 50% chance of winning 200 Naira vs. 50% chance of winning 200 Naira; (2) 50% chance of winning 100 Naira vs. 50% chance of winning 300 Naira; and (3) 50% chance of winning 0 Naira vs. 50% chance of winning 400 Naira. Experimental designs for measuring risk preferences require subjects to have an understanding of probabilistic events and calculations and are likely to be more challenging for less-educated populations [27]. Because we were unable to assess numeracy skills, choices in the lottery games were simplified so that the probabilities of win/loss and expected payouts were constant. While imperfect for mimicking risk aversion, we made these simplifications to isolate only risk preferences associated with the variance in payouts and minimize confounding due to miscalculated probabilities. The winning amount was paid in the form of mobile phone credits. Prior to enrollment, respondents were informed that they would have a chance to gain additional phone credits, but the amounts were not specified.

### Data analysis

Survey data were independently entered into database interface forms using a standardized data dictionary and compared for errors and corrections. A 10% random check was performed to ensure 99.5% data entry accuracy. A second round of quality checks were conducted for all data fields in the codebook to examine outliers and miscodes on an individual case-by-case basis. Where discrepancies were found, data inputs were crosschecked with the original paper survey for final reconciliation. All indeterminate cases were coded to missing.

We first explored the profiles of individuals according to their risk game choice to assess the internal validity of the game measure. We compared basic sociodemographic characteristics—age, sex, martial status, education, employment, and wealth—across game choices by graphing the probability density function of participants’ ages. Differences in the full age profiles of individuals choosing different risk games was tested using the Kruskal-Wallis equality-of-populations rank test. Wealth quintiles were constructed using standard principal components analysis of household asset indicators [28].

We then applied bivariable and multivariable regression analyses to examine relationships between risk game choices, different malaria care-seeking behaviors, and potential mediating pathways. We selected predictors based on *a priori* hypotheses of determinants of health behavior, including unobservable risk preferences *vis-à-vis* the risk game choice and observable sociodemographic characteristics. We then chose two behavioral outcomes that were statistically significant from bivariable analyses—having ever had a blood test for malaria, and waiting three or more days before seeking care for the current illness—were then chosen for further multivariable analysis. We then introduced observable sociodemographic characteristics—demographic profile, education, and wealth—individually and separately to examine the contributions of these factors for mediating the relationship between risk game choices and behavioral outcomes. Interaction effects between risk game choices and education and wealth indicators were explored, but not reported because of the limited sample size. We used logistic regressions for all outcome measures, standard errors were clustered by drug retail site, and Wald statistics are reported for each regression equation. P-values for Wald tests of joint significance are also reported for categorical variables. All data analysis was conducted using Stata MP version 12.

### Ethical considerations

The Nigerian Health Research Ethical Review Committee and the University of California, San Francisco’s Committee for Human Research approved all study protocols. Surveyors obtained informed consent from study participants and shop proprietors where the customers were recruited. Funding sponsors for the study did have any role in the study design, execution, or publication.

## Results

**Figure 1:**
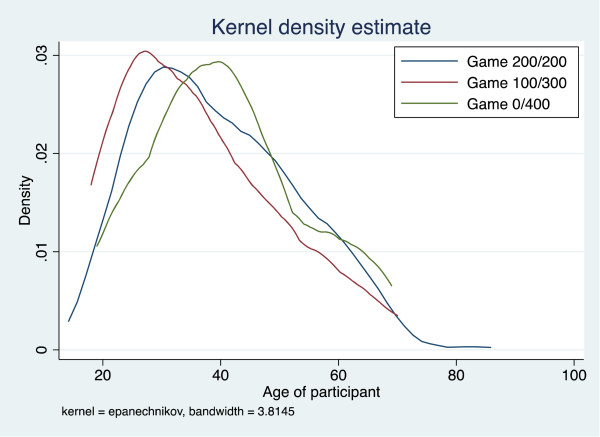
**Kernel density distribution of participants’ ages by risk game chosen.** Legend: Compared to 200/200 Naira group (blue), individuals choosing the 0/400 Naira game (green) were slightly older and those choosing the 100/300 Naira game (red) were generally younger; the full age profile of the 0/400 Naira group lies everywhere to the right of the 100/300 Naira group. Age distributions across risk games were significantly different (p = 0.015), tested using the Kruskal-Wallis equality-of-populations rank test.

Of 448 individuals who agreed to participate in the risk game, 63.2% (n = 283) chose the game with a certain payout of 200 Naira, 27.9% (n = 125) chose the game with a chance of either 100 or 300 Naira payout, and 8.9% (n = 40) chose the game with the largest payout variance, 0 or 400 Naira (see Table 1). Individuals choosing different game choices were marginally significantly different by sex and education, and significantly different by age. Nearly 68% of individuals choosing the 0/400 Naira game were male versus only 45.6% of those choosing the 100/300 Naira game and 50.2% of those choosing the 200/200 Naira game. Compared to 200/200 Naira group, individuals choosing the 0/400 Naira game were slightly older and those choosing the 100/300 Naira game were generally younger; the full age profile of the 0/400 Naira group lies everywhere to the right of the 100/300 Naira group (see Figure 1). The 200/200 Naira group was somewhat better educated than others with only 19.4% having primary schooling or less compared with 29.6% and 25.0% of the 100/300 Naira and 0/400 Naira groups, respectively. There were statistically significant differences in employment status across risk game groups: 50.7% of the 200/200 Naira group was self-employed compared to 59.2% and 57.5% of the 100/300 Naira and 0/400 Naira groups, respectively.

**Table 1:** **Sample characteristics**

		Sample overall	Risk game chosen			
			200/200	100/300	0/400	***p***for diff
Sex	% Male	50.4	50.2	45.6	67.5	*
Age^1^	Median age	36.4	38.0	33.0	40.0	**
	CI	[20.0-63.0]	[21.0-63.0]	[19.0-64.0]	[22.0-64.0]	
Marital status	% Married	67.4	67.8	64.0	75.0	
Education	% Less than primary	8.5	7.4	8.8	15.0	*
	% Primary	14.3	12.0	20.8	10.0	
	% Secondary	39.3	39.2	40.8	35.0	
	% More than secondary	37.9	41.3	29.6	40.0	
Employment status^2^	% Employed full time	28.2	30.5	23.2	27.5	***
	% Employed part time	2.9	3.9	1.6	0.0	
	% Self-employed	53.7	50.7	59.2	57.5	
	% Unemployed	15.2	14.9	16.0	15.0	
Wealth	% Poorest	19.6	18.4	19.2	30.0	
	% Poorer	20.3	18.4	24.8	20.0	
	% Middle	19.6	20.1	19.2	17.5	
	% Richer	20.1	21.9	16.8	17.5	
	% Richest	20.3	21.2	20.0	15.0	
N		448	283	125	40	
	% Chosen		63.2	27.9	8.9	

95% Confidence intervals (CI) in brackets.

*** p < 0.01, ** p < 0.05, * p < 0.10.

^1^Age distribution differences tested by the Kruskal-Wallis equality-of-populations rank test (p = 0.015).

^2^ *N* = 447 due to 1 missing observation.

Differences in usual malaria care-seeking practices and specifically for the current illness are summarized in Table 2. Testing for malaria is generally low: 61.4% have ever had a blood test and 22.8% report that testing or examinations are part of their usual malaria care-seeking behavior. More individuals in the 0/400 Naira group reported having ever had a blood test for malaria (74.4% compared to 52.1% of the 100/300 Naira group and 63.7% of the 200/200 Naira group), and these differences were statistically different. While other usual malaria care-seeking behaviors were not significantly different, some trends across risk game groups were observed: a higher percentage of individuals in the 0/400 Naira group reported being completely disabled (defined as “not being able to perform any normal activities”) and obtaining care at a PPMV during their last malaria episode than among other risk game groups.

**Table 2:** **Differences in malaria care-seeking and treatment behavior by risk game**

			Risk game chosen			
	N	Sample overall	200/200	100/300	0/400	***p***for diff
Usual malaria care-seeking behavior						
% Ever had blood test for malaria	438	61.4	63.7	52.1	74.4	***
% Usually gets diagnosed with a test	448	22.8	23.3	22.4	20.0	
% Usually diagnosed via provider consult	448	31.5	29.0	36.8	32.5	
% Was completely disabled due to illness last time had malaria	436	16.7	15.9	16.4	23.7	
% Saw PPMV last time suspected malaria	422	41.5	39.6	42.5	51.4	
Care-seeking behavior for current illness						
Waited 3 or more days before seeking care	414	34.8	34.5	40.5	18.9	**
% Diagnosed by myself/family/friend	447	91.5	90.8	92.7	92.5	
% Bought an ACT	410	44.4	43.7	44.5	48.6	
% Bought an additional symptom drug	417	61.9	61.9	61.3	63.2	
% RDT-positive	448	4.0	4.6	3.2	2.5	
Mean amount paid for drugs	434	381.7	396.7	353.1	366.3	
CI		[341.6-421.9]	[342.7-450.8]	[291.9-414.4]	[25.96-516.6]	
Treatment for current illness^1^						
Followed treatment directions^2^	407	73.2	73.8	74.5	64.9	
Took non-malaria drugs^3^	255	77.6	77.8	76.8	79.2	
Increased willingness to pay for an RDT from baseline	403	12.2	12.9	12.8	5.3	
Baseline mean (Naira)	403	402.7	396.9	410.6	419.7	
CI		[390.1-415.4]	[380.8-413.0]	[386.5-434.6]	[378.5-461.0]	
Follow-up mean (Naira)	403	405.6	401.6	416.1	402.6	
CI		[393.3-417.9]	[385.8-417.3]	[393.2-439.9]	[361.6-445.7]	

95% Confidence interval in brackets.

*** p < 0.01, ** p < 0.05, * p < 0.10.

^1^ Restricted to those who were followed up by phone.

^2^ Defined as taking anti-malarial drugs if RDT-positive and not taking anti-malarial drugs if RDT-negative.

^3^ Restricted to those who purchased non-malaria drugs.

160 Naira is equivalent to about US$1.00.

For the current illness, overall, 34.8% of participants waited three or more days before seeking care; the percentage was significantly lower for those in the 0/400 Naira group (18.9%) and higher for those in the 100/300 Naira group (40.5%). Self-diagnosis is generally high and not different across risk game groups. No differences in drug purchases, amount paid, and RDT-positivity were observed. While differences in RDT acceptability measures were also not detected, fewer individuals in the 0/400 Naira group (64.9%) followed RDT result-specific treatment directions compared to the 100/300 Naira group (74.5%) and the 200/200 Naira group (73.2%). In terms of willingness to pay for an RDT, fewer individuals in the 0/400 Naira group (5.3%) reported an increase in their willingness to pay for an RDT compared to other risk game groups (12.8% among the 100/300 group and 12.9% among the 200/200 group). These changes were largely driven by a decline in mean willingness to pay between baseline and follow-up within the 0/400 Naira group. In contrast, mean willingness to pay increased over time for the other risk game groups.

Multivariable regression analyses that investigate possible associations between risk preferences and health behaviors were statistically significant in bivariable analyses (i.e. having ever had a blood test, waiting three or more days before seeking care) are displayed in Tables 3 and 4. For having ever had a blood test for malaria (Table 3), the point estimates on risk game choices do not substantively change across specifications; this suggests that there is little confounding from the additional controls. When additional sociodemographics variables are added, risk game choices are jointly significant for most specifications with marginal joint significance showing when education is controlled for (column 3). In column 2, the significantly lower likelihood associated with choosing the 100/300 Naira game becomes marginally significant when demographic variables are added. Choosing the game with the highest variance (0/400 Naira) is associated with a higher, but not statistically significant likelihood of ever having a blood test. Married individuals and those from Ibadan are nearly twice as likely as their unmarried or Ogbomosho counterparts to ever have had a blood test. With education added (column 3), neither risk game choice is statistically significant. Education alone is highly predictive of increased likelihood of testing by more than three-fold. When wealth measures are separately included (column 4), the estimated effects of risk game choices are similar, are individually marginally significant, and are jointly significant. Estimates on wealth measures indicate a steep gradient with the wealthiest being five times more likely to have ever been tested. Education and wealth remain significant predictors when all controls are added together (column 5).

**Table 3:** **Logistic regressions predicting the likelihood of ever having a blood test for malaria (odds ratios)**

	Ever had blood test for malaria				
	(1)	(2)	(3)	(4)	(5)
Risk game (reference: Game 200/200)					
Game 100/300	0.620**	0.651*	0.736	0.668*	0.728
	[0.393 - 0.977]	[0.406 - 1.045]	[0.459 - 1.180]	[0.415 - 1.076]	[0.449 - 1.180]
Game 0/400	1.655	1.779	1.978	2.073*	2.112*
	[0.722 - 3.794]	[0.726 - 4.362]	[0.790 - 4.957]	[0.937 - 4.587]	[0.922 - 4.834]
*Joint test p-value*		*0.039*	*0.070*	*0.020*	*0.038*
Male (reference: female)		0.890	0.808	0.797	0.772
		[0.606 - 1.308]	[0.531 - 1.231]	[0.527 - 1.204]	[0.500 - 1.194]
Age (reference: 18–29)					
30-39		0.942	0.913	1.003	0.960
		[0.522 - 1.699]	[0.519 - 1.608]	[0.549 - 1.832]	[0.539 - 1.712]
40-49		1.338	1.373	1.388	1.382
		[0.628 - 2.851]	[0.638 - 2.956]	[0.649 - 2.969]	[0.636 - 3.004]
50+		1.590	2.063**	1.776**	2.031**
		[0.886 - 2.853]	[1.134 - 3.755]	[1.004 - 3.141]	[1.100 - 3.751]
*Joint test p-value*		*0.081*	*0.008*	*0.033*	*0.019*
Married (reference: not married)		1.777**	1.840**	1.665*	1.735*
		[1.079 - 2.927]	[1.070 - 3.164]	[0.981 - 2.826]	[0.992 - 3.034]
Recruited at PPMV (reference: pharmacy)		0.707	0.847	0.835	0.908
		[0.454 - 1.101]	[0.510 - 1.407]	[0.512 - 1.363]	[0.531 - 1.552]
Ibadan (reference: Ogbomosho)		2.181***	2.192***	1.561*	1.757**
		[1.406 - 3.382]	[1.345 - 3.574]	[0.945 - 2.580]	[1.020 - 3.027]
Education (reference: less than primary)					
Primary			1.105		1.068
			[0.478 - 2.554]		[0.484 - 2.360]
Secondary			2.017		1.539
			[0.774 - 5.254]		[0.636 - 3.720]
Higher			3.656***		2.413**
			[1.473 - 9.077]		[1.042 - 5.591]
*Joint test p-value*			*0.012*		*0.040*
Wealth (reference: poorest quintile)					
Second				1.562	1.200
				[0.876 - 2.783]	[0.690 - 2.085]
Third				1.992**	1.571
				[1.026 - 3.866]	[0.868 - 2.843]
Fourth				2.742**	1.836*
				[1.244 - 6.043]	[0.905 - 3.728]
Richest				5.156***	3.304***
				[2.633 - 10.098]	[1.781 - 6.129]
*Joint test p-value*				*0.000*	*0.003*
*N*	438	438	438	438	438
*Wald chi2 (df)*	9.4 (2)	106.1 (9)	86.2 (12)	123.5 (13)	140.4 (16)
*p*	<0.01	<0.001	<0.001	<0.001	<0.001

Standard errors are clustered at the shop level.

95% Confidence intervals in brackets.

*** p < 0.01, ** p < 0.05, * p < 0.10.

p-values for Wald statistics reported for joint tests.

**Table 4:** **Logistic regressions predicting the likelihood of waiting three or more days before seeking care (odds ratios)**

	Waited 3 days or more before seeking care				
	(1)	(2)	(3)	(4)	(5)
Risk game (reference: Game 200/200)					
Game 100/300	1.294	1.434	1.519	1.416	1.538*
	[0.828 - 2.023]	[0.879 - 2.339]	[0.919 - 2.511]	[0.874 - 2.292]	[0.929 - 2.545]
Game 0/400	0.443*	0.512	0.521*	0.487*	0.458**
	[0.194 - 1.011]	[0.230 - 1.141]	[0.246 - 1.103]	[0.221 - 1.075]	[0.227 - 0.923]
*Joint test p-value*		*0.064*	*0.045*	*0.050*	*0.018*
Male (reference: female)		0.595*	0.565**	0.608*	0.577**
		[0.349 - 1.015]	[0.334 - 0.958]	[0.364 - 1.017]	[0.347 - 0.961]
Age (reference: 18–29)					
30-39		0.945	0.929	0.946	0.905
		[0.526 - 1.698]	[0.517 - 1.669]	[0.526 - 1.702]	[0.514 - 1.594]
40-49		1.110	1.140	1.119	1.148
		[0.597 - 2.062]	[0.616 - 2.107]	[0.558 - 2.245]	[0.591 - 2.231]
50+		1.558	1.714	1.597	1.906*
		[0.869 - 2.791]	[0.880 - 3.336]	[0.803 - 3.176]	[0.919 - 3.951]
*Joint test p-value*		*0.196*	*0.188*	*0.268*	*0.092*
Married (reference: not married)		1.311	1.209	1.328	1.269
		[0.886 - 1.941]	[0.823 - 1.775]	[0.890 - 1.980]	[0.890 - 1.810]
Recruited at PPMV (reference: pharmacy)		0.907	0.994	0.883	0.952
		[0.605 - 1.360]	[0.646 - 1.529]	[0.570 - 1.368]	[0.594 - 1.527]
Ibadan (reference: Ogbomosho)		3.640***	3.657***	3.907***	4.481***
		[1.665 - 7.957]	[1.692 - 7.900]	[1.676 - 9.112]	[1.889 - 10.627]
Education (reference: less than primary)					
Primary			2.586**		2.847***
			[1.216 - 5.499]		[1.303 - 6.220]
Secondary			1.830		2.498*
			[0.730 - 4.585]		[0.970 - 6.434]
Higher			3.190***		5.113***
			[1.470 - 6.923]		[2.021 - 12.936]
*Joint test p-value*			*0.008*		*0.003*
Wealth (reference: poorest quintile)					
Second				0.861	0.658
				[0.300 - 2.470]	[0.224 - 1.932]
Third				1.034	0.804
				[0.451 - 2.369]	[0.346 - 1.869]
Fourth				0.606	0.362*
				[0.236 - 1.556]	[0.123 - 1.067]
Richest				0.863	0.477
				[0.424 - 1.754]	[0.189 - 1.202]
*Joint test p-value*				*0.410*	*0.172*
*N*	414	414	414	414	414
*Wald chi2 (df)*	6.5 (2)	48.9 (9)	64.3 (12)	51.8 (13)	78.3 (16)
*p*	<0.05	<0.001	<0.001	<0.001	<0.001

Standard errors are clustered at the shop level.

95% Confidence intervals in brackets.

*** p < 0.01, ** p < 0.05, * p < 0.10.

p-values for Wald statistics reported for joint tests.

When examining days waited before seeking care (Table 4), the joint significance of risk game choices fluctuates around the 5% significance level across difference specifications and the addition of education and wealth measures also does not substantively change the individual point estimates. With just demographic controls (column 2), neither risk category is statistically significant, but being female and residing in Ibadan is associated with a higher likelihood of waiting at least three days. Education is also associated with a higher likelihood of waiting longer (column 3), but wealth does not appear to have an independent effect (columns 4–5). In the final specification with the full set of controls, the point estimates on risk game and education variables increase as do statistical significance levels. In sensitivity analyses (not shown), polynomial age and employment status variables added to regression specifications were neither statistically or substantively significant.

## Discussion

From a pilot study of malaria case management at drug vendors in Oyo State, Nigeria, we find suggestive evidence that a fundamental trait, risk preferences, may be influencing individuals’ behaviors in treating suspected malaria. With the offering of three simplified games with constant expected values but different payout variances, nearly two-thirds of respondents chose the game without any risk and less than 10% chose the game with the highest payout variance. A comparison of basic demographics between risk profiles suggests that our measure of risk preference may be valid and in line with conventional findings (e.g. [29]): individuals choosing games with some variance in payouts were more likely to be male, self-employed, and less educated compared to those choosing games with a certain payout. Contrary to usual findings from developed countries (e.g. [30]), respondents choosing the “riskiest” game with the highest payout variance were generally older than their less risky counterparts. This divergence may be related to cultural perceptions of seniority and rank, but further qualitative analysis is needed to better characterize this finding and a larger sample size is needed to better assess the external validity of the risk game measure.

According to RDT results, respondents did not differ by malaria burden across risk groups, but they approached care-seeking in somewhat different ways. While a number of malaria care-seeking and treatment behaviors were not statistically significantly different across risk groups, the direction of the differences tend to align and point to two divergent ways for approaching healthcare. Analysis of risk game profiles in relation to health behaviors suggest that individuals who prefer less risk *vis-à-vis* their game choice tended to be more “conservative” while those who preferred more risk tended to be less cautious. From bivariable analyses, respondents choosing the least risky, certain payout game were more likely to ever have had a blood test, and less likely to visit PPMVs, report fewer debilitating effects of malaria, and seek care later. In particular, the “riskiest” group choosing the highest variance game appears to be a highly select group of individuals who may have more health experiences, interactions with the health care sector, or have more health problems in general, such as due to natural aging, but even after controlling for age, these differences in behavior remained.

However, not all behaviors accord perfectly with this simplification across risk groups; the behavior of individuals in the moderate variance (i.e. 100/300 Naira) group sometimes aligned more with the no variance group (i.e. 200/200 Naira) and sometimes aligned more closely with those in the highest variance group (i.e. 0/400 Naira). This was also evident in multivariate analyses in which the direction for the effect for the high-risk (0/400) group differed from the moderate risk group (100/300) when compared to the no risk group (200/200) group. The differences were only sometimes statistically significant, suggesting that the moderate risk individuals may sometimes display similar intolerance (i.e. when not significantly different) or even more intolerance (i.e. when significantly different) for risk as the no risk group. Better differentiation and characterization of this moderate group is needed and with potentially different game payout structures.

Poorer or less educated individuals are generally thought to have lower health status, face greater barriers to accessing health services, and be subject to tighter income constraints. In the Nigerian context, PPMVs provide lower quality drugs and care than pharmacists and disproportionately serve the poor (and potentially sicker) populations [20, 31]. Education was a strong predictor for both behaviors investigated and a strong wealth gradient was observed for the likelihood of ever having had a blood test for malaria. Even though these socioeconomic status indicators were predictive of certain behaviors, point estimates of the effects of risk game choices remained substantively unchanged, even after controlling for various sets of confounders, suggesting possible independent effects of unobservable risk preferences.

One unique contribution of this study is that risk preferences are evaluated in relation to the introduction of a health device, the uptake of new information, and the implications of diagnostic certainty on actual treatment behaviors. Although results are not statistically significant for any treatment outcomes (owing to the small sample size), the directionality of the observed relationships suggest that individuals of varying risk types may approach health information differently, at least among those seeking care for suspected malaria in our sample. Compared to individuals in the “riskiest” group with less cautious care-seeking behavior, a higher proportion of those in the zero- and low-variance (i.e. 200/200 and 100/300 Naira) risk groups reported following the correct treatment procedure and an increase in willingness to pay for an RDT. This may be related to a greater understanding or learning of the value of diagnostic testing over the course of illness recovery. In fact, individuals in the riskiest group reduced their willingness to pay for the RDT over time, suggesting that they value the diagnostic information less and do not view the RDTs to be as a useful a device as others. Health promotion for RDTs should bear these harder-to-convince individuals in mind when designing and framing messages. While the overall high rate of treatment adherence to RDT results indicates generally high acceptability of RDTs, it is unclear if introducing RDTs would decrease presumptive treatment over the long-term. Studies elsewhere show that reduced misdiagnosis and positive experiences with health products can foster stronger learning effects and increase adoption.^c^

These results should be interpreted in light of several caveats. Because of the targeted recruitment at drug shops, the study population is not a representative sample of all adults with suspected malaria, limiting the broader generalizability of these findings. Within our sample, levels of reported willingness to pay should not be taken out of context and used to infer market pricing, as individuals’ responses were not elicited for that purpose. Further, in our analysis, there may be other unobserved traits that explain differences in care-seeking behaviors. For example, rather than being a riskier person, individuals might be time inconsistent and seek immediate gratification for the condition, especially when sick. In a companion paper, we find a significant effect of an SMS reminder message of RDT results to “nudge” participants to overcome time inconsistency and follow the correct treatment, but there was no additional interaction with risk game choices [25]. Future work should seek to additionally assess the contributions of time preferences, the initial state of health (sick versus well), and include a larger game choice set with graduated payouts to better characterize risk aversion. In particular, we find only 8.9% of our sample took up the riskiest game. These individuals may have another unobserved common trait, such as numeracy, which is related to both their risk preference and health behaviors. Risk preferences will be included in a larger study of adoption of RDTs to test whether these same relations emerge. Estimates from this pilot study will serve as the basis for sample size calculations of the expanded study that will enable a more thorough multivariable analysis of risk preferences. Finally, it is important to note that all health outcome measures are self-reported and that there may be important mediating factors, such as where and from whom participants received advice on appropriate malaria diagnosis and treatment, which may influence care-seeking behaviors. Future studies should seek to assess the contributions of these factors independent from an individual’s fundamental risk preferences.

## Conclusion

While more research is needed to explain care-seeking behaviors of individuals with different preferences and traits, the main findings of this exploratory study suggests that risk preferences may independently influence the way individuals process health information from a newly introduced health technology. Communications designed to change behavior often ignore differences in the uptake and processing of health advice. Messages delivered through either provider-patient interactions or targeted social mobilization can include multiple message frames in order to reach individuals with varying preferences. Different modalities need to be tested to assess what decision frame resonates most with different groups. In particular, crafting messages that appeal to the unique concerns of different risk types would increase the reach of interventions, and specifically in this case, work to enhance the adoption of RDTs and increase the demand for testing before treatment.

### Endnotes

^a^Tarozzi A, Mahajan A, Blackburn B, Kopf D, Krishnan L, and Yoong J: Micro-loans, bednets and malaria: Evidence from a randomized controlled trial in Orissa (India). American Economic Review 2014, 104(7), 1909-1941. Cohen, J., Dupas, P., & Schaner, S. (2013). Price subsidies, diagnostic tests, and targeting of malaria treatment: Evidence from a randomized controlled trial. NBER Working Paper No. 17943. Available online at http://www.nber.org/papers/w17943. Accessed on March 20, 2013.

^b^See Cohen, J., Dupas, P., & Schaner, S. (2013). Price subsidies, diagnostic tests, and targeting of malaria treatment: Evidence from a randomized controlled trial. NBER Working Paper No. 17943. Available online at http://www.nber.org/papers/w17943. Accessed on March 20, 2013.

^c^See Adhvaryu, A. R. (2012). Learning, misallocation, and technology adoption: Evidence from new malaria therapy in Tanzania (available online at http://papers.ssrn.com/sol3/papers.cfm?abstract_id=1357771) and Dupas, P. (2012). Short-run subsidies and long-run adoption of new health products: Evidence from a field experiment. CEGA Working Paper Series No. 011 (available online at http://escholarship.org/uc/cega_wps)

## Abbreviations

ACT: Artemisinin-based combination therapy
AMFm: Affordable medicines facility - malaria
CI: Confidence interval
RDT: Rapid diagnostic test
PPMV: Proprietary and patent medicine vendors
SMS: Short message service.
